# Using Cluster Analysis to Overcome the Limits of Traditional Phenotype–Genotype Correlations: The Example of *RYR1*-Related Myopathies

**DOI:** 10.3390/genes14020298

**Published:** 2023-01-23

**Authors:** Claudia Dosi, Anna Rubegni, Jacopo Baldacci, Daniele Galatolo, Stefano Doccini, Guja Astrea, Angela Berardinelli, Claudio Bruno, Giorgia Bruno, Giacomo Pietro Comi, Maria Alice Donati, Maria Teresa Dotti, Massimiliano Filosto, Chiara Fiorillo, Fabio Giannini, Gian Luigi Gigli, Marina Grandis, Diego Lopergolo, Francesca Magri, Maria Antonietta Maioli, Alessandro Malandrini, Roberto Massa, Sabrina Matà, Federico Melani, Sonia Messina, Andrea Mignarri, Maurizio Moggio, Elena Maria Pennisi, Elena Pegoraro, Giulia Ricci, Michele Sacchini, Angelo Schenone, Simone Sampaolo, Monica Sciacco, Gabriele Siciliano, Giorgio Tasca, Paola Tonin, Rossella Tupler, Mariarosaria Valente, Nila Volpi, Denise Cassandrini, Filippo Maria Santorelli

**Affiliations:** 1 IRCCS Fondazione Stella Maris, 56128 Pisa, Italy; 2 Kode Data Analysis s.r.l., 56128 Pisa, Italy; 3 IRCCS C. Mondino Foundation, 27100 Pavia, Italy; 4 Center of Translational and Experimental Myology, IRCCS Istituto Giannina Gaslini, 16147 Genova, Italy; 5 Department of Neuroscience, Rehabilitation, Ophthalmology, Genetics, Maternal and Child Health—DINOGMI, University of Genova, 16147 Genova, Italy; 6 Department of Advanced Medical and Surgical Sciences, University of Campania “Luigi Vanvitelli”, 81100 Naples, Italy; 7 Dino Ferrari Center, Department of Pathophysiology and Transplantation, University of Milan, 20122 Milan, Italy; 8 Foundation IRCCS Ca’ Granda Ospedale Maggiore Policlinico, Neurology Unit, 20122 Milan, Italy; 9 Metabolic Disease Unit, AOU Meyer Children Hospital, 50139 Florence, Italy; 10 Unit of Neurology and Neurometabolic Diseases, Department of Medical, Surgical and Neurological Sciences, University of Siena, Viale Bracci 2, 53100 Siena, Italy; 11 Department of Clinical and Experimental Sciences, University of Brescia, NeMO-Brescia Clinical Center for Neuromuscular Diseases, 25064 Brescia, Italy; 12 Neurology Unit, Department of Neurosciences, University Hospital of Udine, 33100 Udine, Italy; 13 Department of Medicine, University of Udine, 33100 Udine, Italy; 14 IRCCS Ospedale Policlinico San Martino, 16132 Genova, Italy; 15 Centro Sclerosi Multipla, ASL Cagliari, 09047 Cagliari, Italy; 16 Neuromuscular Diseases Unit, Department of Systems Medicine, Tor Vergata University of Rome, 00133 Rome, Italy; 17 Careggi University Hospital, Neurology Unit, 50134 Florence, Italy; 18 Pediatric Neurology, AOU Meyer Children Hospital, 50139 Florence, Italy; 19 Unit of Neurology and Neuromuscular Disorders, Department of Clinical and Experimental Medicine, University of Messina, 98122 Messina, Italy; 20 Foundation IRCCS Ca’ Granda Ospedale Maggiore Policlinico, Neuromuscular and Rare Diseases Unit, Department of Neuroscience, 20122 Milan, Italy; 21 Neuromuscular Diseases Center, Neurology Unit, San Filippo Neri Hospital, 00135 Rome, Italy; 22 Department of Neurosciences, University of Padova, 35122 Padova, Italy; 23 Department of Clinical and Experimental Medicine, University of Pisa, 56126 Pisa, Italy; 24 Unit of Neurology, Fondazione Policlinico Universitario A. Gemelli IRCSS, 00168 Rome, Italy; 25 John Walton Muscular Dystrophy Research Centre, Newcastle University and Newcastle Hospitals NHS Foundation Trusts, Newcastle upon Tyne NE1 3BZ, UK; 26 Department of Neuroscience, Biomedicine and Movement Sciences, University of Verona, 37129 Verona, Italy; 27 Department of Biomedical, Metabolic and Neural Sciences, University of Modena and Reggio Emilia, 41121 Modena, Italy; 28 Department of Molecular Cell and Cancer Biology, University of Massachusetts Medical School, Worcester, MA 01655, USA

**Keywords:** *RYR1*-related myopathies, unsupervised cluster analysis, NGS, genotype–phenotype correlation

## Abstract

Thanks to advances in gene sequencing, *RYR1*-related myopathy (RYR1-RM) is now known to manifest itself in vastly heterogeneous forms, whose clinical interpretation is, therefore, highly challenging. We set out to develop a novel unsupervised cluster analysis method in a large patient population. The objective was to analyze the main *RYR1*-related characteristics to identify distinctive features of RYR1-RM and, thus, offer more precise genotype–phenotype correlations in a group of potentially life-threatening disorders. We studied 600 patients presenting with a suspicion of inherited myopathy, who were investigated using next-generation sequencing. Among them, 73 index cases harbored variants in *RYR1*. In an attempt to group genetic variants and fully exploit information derived from genetic, morphological, and clinical datasets, we performed unsupervised cluster analysis in 64 probands carrying monoallelic variants. Most of the 73 patients with positive molecular diagnoses were clinically asymptomatic or pauci-symptomatic. Multimodal integration of clinical and histological data, performed using a non-metric multi-dimensional scaling analysis with k-means clustering, grouped the 64 patients into 4 clusters with distinctive patterns of clinical and morphological findings. In addressing the need for more specific genotype–phenotype correlations, we found clustering to overcome the limits of the “single-dimension” paradigm traditionally used to describe genotype–phenotype relationships.

## 1. Introduction

The higher-than-expected rates of pleiotropy and allelic heterogeneity in hereditary myopathies and muscular dystrophy that are emerging in the era of next-generation sequencing (NGS) are major impediments to precise patient stratification and the development of precision medicine treatments [1]. The “one gene-one phenotype” paradigm is now being challenged by the discovery of situations where a single gene is linked to several conditions or, vice versa, a single disease has multiple possible culprit genes. Such situations are common outputs of the use of targeted multigene panels and exome sequencing platforms in clinical practice. The large datasets handled using these methods and the frequently poor correlations between the predicted effects of most variants and the actual disease manifestations result in high levels of uncertainty in clinical diagnosis and reduce the translational value of such “big data” generation [2]. It has become fundamental to incorporate advanced analytics into the practice of translational medicine to fully exploit the complete range of diagnostic data that may be yielded by a single individual.

The skeletal muscle ryanodine receptor gene (*RYR1*) is a large gene encoding ryanodine receptor 1, a 565 kDa homotetrameric calcium ion release channel playing a pivotal role in Ca^2+^ intracellular signaling and excitation-contraction coupling [3,4]. *RYR1* is associated with multiple, apparently diverse, clinical conditions, including inherited malignant hyperthermia (MIM 145600), central core disease (MIM 11700), and multi-minicore disease (MIM 255320)—both muscle diseases, centronuclear myopathy (MIM 255320), and congenital fiber-type disproportion (MIM 255310) [5,6]. However, these typical histopathological features are not unique to *RYR1*-related disorders, are variable over time, and often non-specific patterns such as type 1 fiber predominance are described [3].In recent years, the association of *RYR1* variants with additional phenotypes (including King–Denborough syndrome, exercise-induced rhabdomyolysis, lethal multiple pterygium syndrome, adult-onset distal myopathy, atypical periodic paralysis with or without myalgia, mild calf-predominant myopathy, and “dusty core” disease) has progressively increased the overlap between the various diagnostic categories [3]. With the continuing emergence of new clinical subtypes along the *RYR1*-related disease spectrum—investigations of unselected cohorts with inherited muscle disorders have even detected adult-onset phenotypes [1], nuanced nomenclatures are being adopted. Moreover, the poor significance and difficult clinical interpretation of many genetic variants are limitations that still need to be overcome. Indeed, even when adopting the strict American College of Medical Genetics and Genomics criteria [7] and considering the reduced penetrance of some alleles [8], differentiating between pathogenic alleles and variants of uncertain significance can be difficult. Moreover, muscle biopsy or myo-imaging studies often show non-specific patterns, with the result that the significance of gene variants in the setting of muscle pathology and muscle weakness remains uncertain [9].

Muscular disease subtype identification is crucial to gaining useful insights into disease pathogenesis and advancing the concept of personalized therapy. Similar to what is seen in complex disorders, such as different forms of cancer [10], we believe that muscular disorders could be better understood using unsupervised data analysis methods allowing a higher level of data integration. With the aim of gathering proof-of-principle evidence in this sense, we used a large cohort of patients with different forms of *RYR1*-related myopathy (*RYR1*-RM) in order to develop a novel unsupervised cluster analysis method. The objective was to analyze the main *RYR1*-related characteristics to identify distinctive features of *RYR1*-RM and, thus, offer more precise genotype–phenotype correlations in a group of potentially life-threatening disorders.

## 2. Methods

### 2.1. Standard Protocol Approvals and Patient Consents

This study was approved by the Tuscany Regional Pediatric Ethics Committee (CEP-InGene2.0 155/2020 (date 3 July 2020)). All of the procedures complied with the Helsinki Declaration of 1975. Genetic studies and muscle biopsies were performed with the written informed consent of patients or, in the case of minors, of patients’ parents or legal guardians.

### 2.2. Patients and Study Design

This is a cross-sectional study that involved 600 patients presenting with a clinical suspicion of inherited myopathy and/or clinical and laboratory findings suggesting a possible diagnosis of neuromuscular disease (NMD) (e.g., muscle weakness, myalgia, fatigability, isolated or pauci-symptomatic hyperCKemia), and referred, over the past five years, to 17 Italian third-level NMD centers for clinical and diagnostic purposes. Gene testing on total genomic DNA was performed in a single laboratory, at IRCCS Fondazione Stella Maris, Pisa, with patients’ written consent. All participants (including parents or legal guardians in the case of children) received pre- and post-testing genetic counseling according to the standard procedure applied at our centers.

In each patient, clinical manifestations were critically appraised by three of the authors (C.D., A.R., C.F.) and digitally coded according to the relative Human Phenotype Ontology (HPO) definitions (https://hpo.jax.org/app/, accessed on 3 November 2022). Magnetic resonance imaging (MRI) scans of thigh and calf muscles, if available, were reviewed by two of the authors (C.F. and G.A.) and taken into consideration when assessing the phenotype. If the skeletal muscle biopsy was performed for diagnostic purposes, three of the authors (A.R., J.B., C.F.) reviewed the morphological features of the sample, histochemical stains, and results of protein studies [11].

Patients were also stratified by the age of onset into the following forms: congenital (onset in the first year of life), childhood (onset before the age of 12 years), juvenile (onset between 12 and 16 years of age), and adult (onset ≥ 16 years). Muscle strength was evaluated using the Medical Research Council (MRC) scores (0–5), both at the first evaluation and during clinical follow-up (which lasted between 1 and 15 years). Creatine kinase (CK) levels were measured at the patients’ latest clinical examination; given the large dataset of CK values, CK levels were categorized as (a) normal (range 0–190 U/L), (b) mildly elevated (<500 U/L), (c) moderately elevated (500–1000 U/L), and (d) high (>1000 U/L).

### 2.3. NGS Workflow and Sequencing Analyses

We used a customized targeted multigene resequencing panel designed using SureDesign technology (Agilent, Santa Clara, CA, USA) and encompassing 241 genes known to be associated with limb-girdle muscular dystrophies and metabolic, congenital, and distal myopathies [12]. A previously validated bioinformatic pipeline [11,13], which adopts the Ingenuity Variant Analysis toolbox (Qiagen, Hilden, Germany), was used for data analysis and variant prioritization in keeping with the guidelines of the American College of Medical Genetics and Genomics [7]. In this study we considered only patients harboring mono/biallelic variants in *RYR1* that have been prioritized using a stringent bioinformatics pipeline and in whom family segregation studies could be performed. To define the impact of missense mutations on protein function, we used an in silico pipeline encompassing the following prediction tools: MutationTaster (http://www.mutationtaster.org/, accessed on 3 November 2022), Mutation Assessor (http://mutationassessor.org/r3/, accessed on 3 November 2022), FATHMM-XF (http://fathmm.biocompute.org.uk/fathmm-xf/, accessed on 3 November 2022), LRT (https://varsome.com/, accessed on 3 November 2022), Deogen2 (https://varsome.com/, accessed on 3 November 2022), Eigen (http://www.columbia.edu/~ii2135/eigen.html/, accessed on 3 November 2022), SIFT4G (https://sift.bii.a-star.edu.sg/sift4g/, accessed on 3 November 2022), Provean (http://provean.jcvi.org/index.php/, accessed on 3 November 2022), MVP (https://varsome.com/, accessed on 3 November 2022), Revel (https://varsome.com/, accessed on 3 November 2022), Primate AI (https://varsome.com/, accessed on 3 November 2022), MetaSVM (https://varsome.com/, accessed on 3 November 2022), MetalR (https://varsome.com/, accessed on 3 November 2022), GERP (https://varsome.com/, accessed on 3 November 2022), PolyPhen-2 HumDir (http://genetics.bwh.harvard.edu/pph2/, accessed on 3 November 2022), PolyPhen-2 HumVar (http://genetics.bwh.harvard.edu/pph2/, accessed on 3 November 2022), UMD Predictor (http://umd-predictor.eu/, accessed on 3 November 2022), and CADD (https://cadd.gs.washington.edu/, accessed on 3 November 2022). Splicing variants and synonymous variants close to splicing sites were also tested using Human Splicing Finder 3.1 (http://www.umd.be/HSF/, accessed on 3 November 2022) and NNSPLICE 0.9 (http://www.fruitfly.org/seq_tools/splice.html/, accessed on 3 November 2022).

To stratify patients by genotype, we first sorted gene variants by their combined annotation-dependent depletion (CADD) score (https://cadd.gs.washington.edu/, accessed on 3 November 2022); CADD is a broadly applicable bioinformatic framework that integrates multiple annotations into one metric in order to prioritize deleterious and disease-causing variants across a wide range of functional categories, effect sizes, and genetic architectures. There is no single universal cut-off value for PHRED-scaled CADD values, but it is common practice in the scientific community to declare variants with a score ≥ 20 “possibly deleterious”. We adopted this approach and sorted *RYR1* variants into two subgroups, namely “likely neutral/benign” (those having CADD scores ≤ 20), and “probably pathogenic” (CADD ≥ 20). We also ascertained that these ranking categories largely matched the conclusions reached when using >50% of the above-mentioned in silico prediction tools [13,14]. Prior to this study, all *RYR1* variants were confirmed by Sanger sequencing, and segregation studies were performed whenever possible.

### 2.4. Western Blot Analysis

Western blot (WB) analyses of muscle biopsy samples were performed as previously described [15], using NuPAGE™ 3–8% Tris-Acetate Mini Gels (Thermo Fisher Scientific, Waltham, MA, USA) to allow separation of large-size molecular weight proteins. Mouse monoclonal anti-ryanodine receptor antibody ab2868 (Abcam, Cambridge, UK) (1:1000) and mouse monoclonal anti-GAPDH antibody ab8245 (Abcam) (1:8000) (as internal standard) were used for this analysis. ImageJ v.1.53a software (https://imagej.nih.gov/ij/, accessed on 29 May 2020) was used for densitometry analyses. Muscle samples from nine healthy individuals were used as controls. The levels of the RYR1 protein in patients grouped in each cluster were expressed in reference to the control mean, which was set to 100%. One-way ANOVA with Dunnett’s multiple comparison test was performed for statistical analysis using Prism version 7.04 (GraphPad Software, La Jolla, CA, USA). Data are shown as mean ± SEM. ** *p* < 0.01, **** *p* < 0.0001.

### 2.5. Cluster Analysis

With the aim of grouping genetic variants and fully exploiting information derived from genetic, morphological, and clinical datasets, we performed unsupervised cluster analysis in probands in whom we were able to: (a) collect detailed data on clinical manifestations and convert them into HPO-based digital codes, (b) review muscle biopsies and transform their findings into binary variables, and (c) assess segregation studies in the family.

The group of patients investigated by NGS included 73 index cases who harbored variants in *RYR1*. Among these, we collected data for this study from only 64 carrying monoallelic variants and different bi-allelic variants whose all clinical and histopathological data were available. To collate complex information into the smallest possible number of dimensions we used a non-metric multi-dimensional scaling (nMDS) algorithm [16,17], an indirect gradient analysis approach that produces an ordination based on a distance or dissimilarity matrix. nMDS seeks to achieve close representation of the pairwise dissimilarity between objects in a low-dimensional space. We also used the Jaccard distance metric for measuring the dissimilarity between datasets [18]. To further explore the results of the nMDS analysis, we used k-means [19], an unsupervised learning method that aims to classify a given dataset through a certain number of clusters fixed a priori. This exploratory analysis constitutes an attempt to identify homogenous groups of cases within the data, if such groups are not previously known, and to disclose non-obvious relationships within the data. Data analysis was performed using the open-source statistical software R and the *vegan* package [20].

## 3. Results

Out of a total of 600 patients with suspicion of NMD consecutively referred from 17 Italian third-level NMD centers to undergo our NGS-based diagnostic workup, 73 (12%) harbored a monoallelic (n = 53) or biallelic (n = 20) variant in *RYR1*; all variants were predicted as disease-associated. The variants in 24 patients were transmitted by an autosomal dominant (AD) pattern of inheritance, while in 47 they arose de novo, and in 2 they were recessively inherited.

### 3.1. Clinical Features

Table S1 summarizes detailed clinical and molecular data of these 73 patients. Most of the cases (63%) were adults (≥16 years) and the sample had a 1.35 M/F ratio (42 males and 31 females). The mean age of onset of the clinical manifestations was 18.0 ± 24.7 years (range 0–72), with 13 cases presenting congenital onset and 20 reporting muscle complaints since childhood. In three patients, the age of onset could not be defined. Data on serum CK levels at the latest neurological examination were available in only 62 patients and showed a broad range (from normal to >2000 UI/L). Among the 73 index cases, 18 (24.6%) had asymptomatic or pauci-symptomatic manifestations consisting of persistent serum CK elevation at rest with or without cramps, myalgia, increased fatigability, and myoglobinuria or episodes of rhabdomyolysis. Histological (n = 64) and WB (n = 22) data from skeletal muscle biopsies were assessed in subsets of the patients.

Exploration of family history showed that 17 index cases (23.3%) with a confirmed genetic diagnosis and an AD pattern of transmission had at least one reportedly affected relative willing to provide clinical and genetic information. Most of these relatives were asymptomatic (20/31, 64.5%); their data are reported in Table S2.

**Figure 1:**
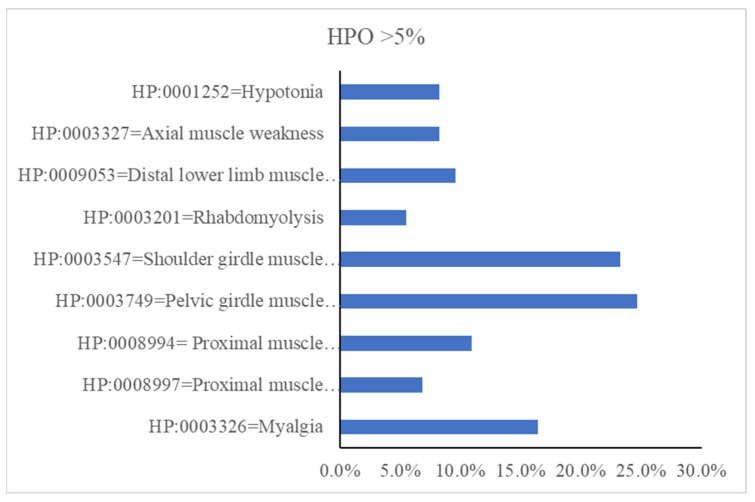
The most representative clinical features of symptomatic patients presented using HPO-based ID codes and nomenclature.

Figure 1 summarizes the distribution of the full set of muscle symptoms in our cohort, grouped by HPO-based codes. Proximal muscle weakness in either the lower (11.0%; HP:0008994) or the upper (6.8%; HP:0008997) limb girdle muscles was the major clinical manifestation in patients harboring variants in *RYR1*. Myalgia (HP:0003326) with or without exercise intolerance was seen in 16.4% of the index cases.

Forty-five patients presented hyperCKemia as the sole or predominant clinical complaint associated with minimal muscle weakness (median Medical Research Council (MRC) Scale score: 5−). Whilst serum CK levels were in the normal range in 17 cases, and 32 patients had mild hyperCKemia (values < 500, normal values < 190 UI/L) or moderately increased CK (values of between 500 and 1000 UI/L, on average three-fold the upper limit of normal), 13 cases repeatedly showed high CK (>1000 UI/L).

Seventeen patients underwent muscle MRI scans at least once during the study. We observed non-specific abnormalities in 12 (70.6%), while the most frequent finding was the presence of discrete fatty infiltration and atrophy of the thigh, gluteus maximus, and medial calf muscles.

In 44/73 index cases, we were able to collect data on multiple clinical evaluations over time (mean follow-up of 3.6 years, range 1–15 years). A comparison of MRC values did not show significant changes in muscle weakness or clinical complaints over time (Figure S1).

Cardiac evaluation, performed in 37 cases, was unremarkable in 25 (67.6%) and showed abnormalities in 12. In the latter subgroup, five patients showed heart rhythm disorders, two had valvular regurgitation, and two presented features suggestive of hypertrophic cardiomyopathy; myocardial infarction, pericarditis, and hypertension were each found in one case.

### 3.2. Muscle Histopathology

We were able to review a total of 64/73 muscle biopsies. Most patients presented one or more of the following features (as shown in Supplementary Tables S1 and S2): an increased number of fibers with internal nuclei (in 26 patients, 40.6%), increased fiber size variation (43 patients, 67.2%), and a mild increase in endomysial connective tissue (7 patients, 10.9%). Undefined myopathic changes were detected in 19 patients (35.8%).

Muscle biopsy of the vastus lateralis demonstrated type 1 fiber predominance [9] in 12 patients. Multiple minicores (n = 12 cases no. 12, no. 18, no. 23, no. 26, no. 32, no. 53, no. 63, no. 64, no. 65, no. 68, no. 69, no. 70) and core-like myopathies (n = 13 cases no. 20, no. 21, no. 22, no. 25, no. 28, no. 30, no. 33, no. 44, no. 45, no. 49, no. 50, no. 54, no. 71) were also observed. Frequently, an increased number of fibers with internal nuclei coexisted with a variation of fiber sizes (n = 23); furthermore, almost half of the patients found to have cores or multiple minicores on histopathological analysis also had this kind of fiber alteration (Supplementary Tables S1 and S2).

**Figure 2:**
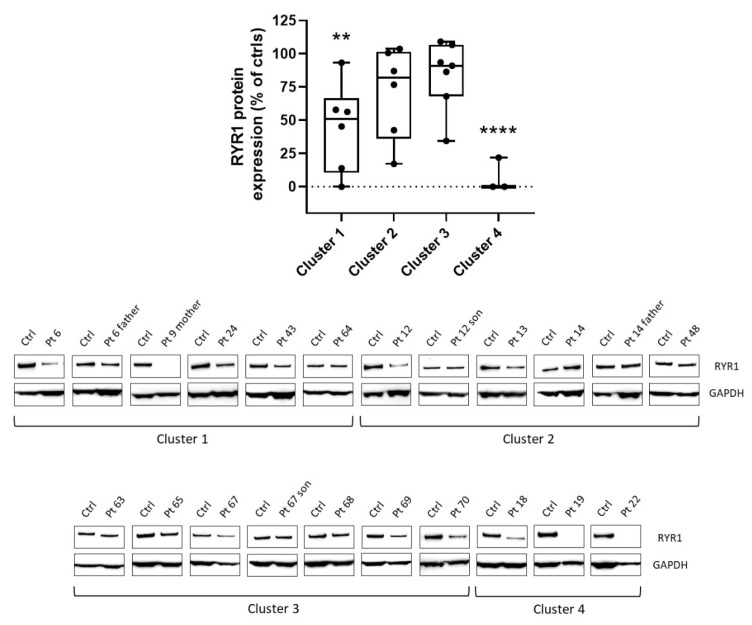
Dots-box plot (top) and representative Western Blot (bottom) show the levels of RYR1 protein in different clusters (expressed as % of controls) and protein detection, respectively. RYR1 protein levels of each cluster were compared to controls (whose levels were set equal to 100%). ** = *p* < 0.05; **** = *p* < 0.0001.

**Figure 3:**
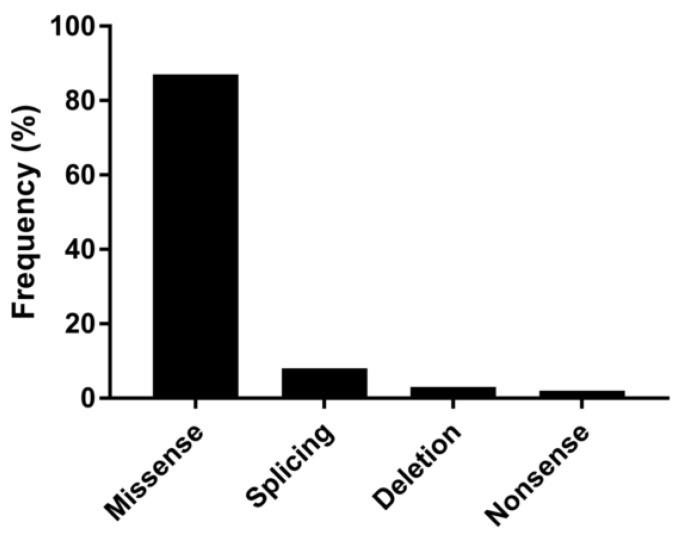
Different mutation types and their relative frequency among the pathogenic ones identified in this study.

Figure 2 shows the results of WB studies performed on 22 patients. Steady-state levels of RYR1 were altered in cluster 1 and severely impaired in cluster 4. More in detail, 12 cases showed <70% of normal levels, of whom six had significantly reduced expression (<50%), and three (cases no. 9m, no. 19, no. 22) almost undetectable expression. Finally, Figure 3 illustrates the spectrum of variant types identified in the index cases. Overall, they were distributed as follows: missense (86.7%), nonsense (2.2%), small in/del variants (3.3%), and variants affecting canonical splice site sequences (7.8%). Interestingly, we observed a possible trend toward a correlation between residual protein expressions in the muscle and severity of gene variants (as defined by CADD scores).

### 3.3. Cluster Analysis

**Figure 4:**
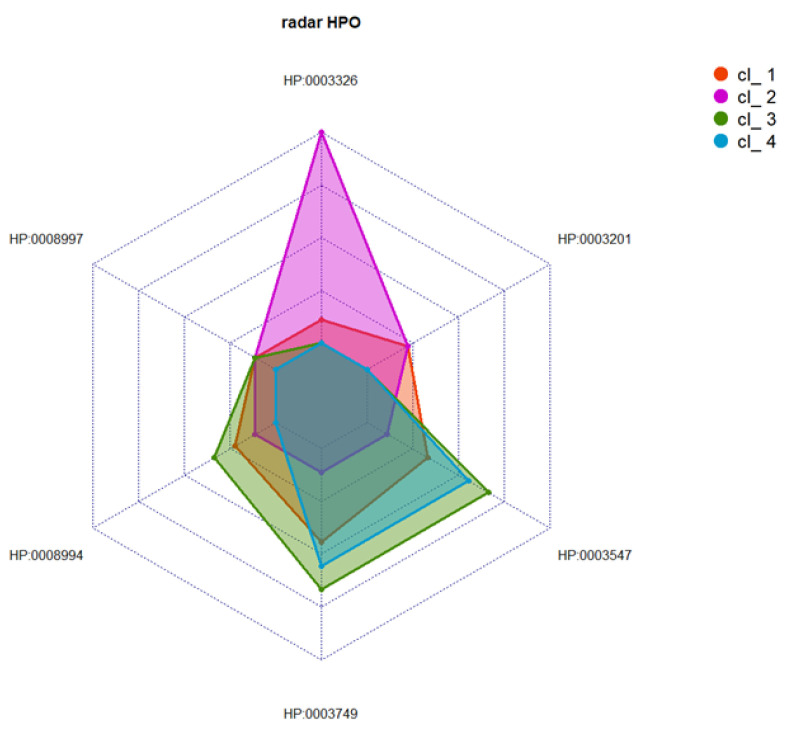
Distribution of frequencies of the most relevant clinical characteristics (indicated as HPO-based codes), shown per cluster.

**Figure 5:**
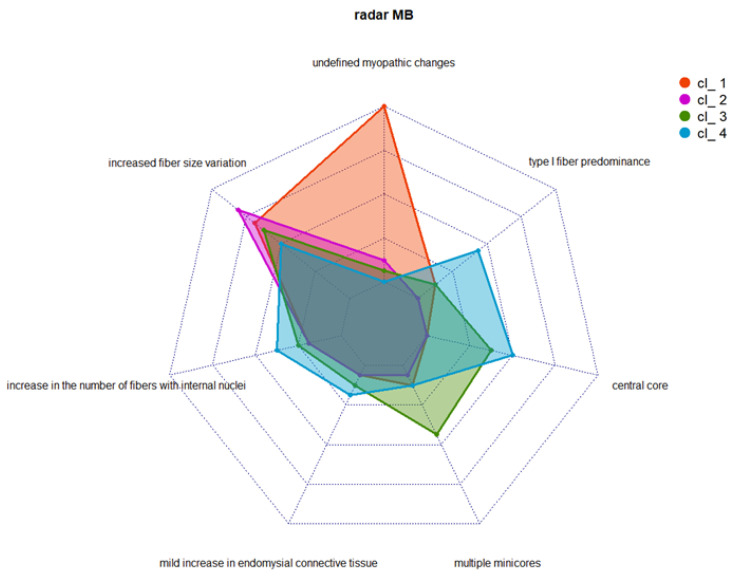
Distribution of frequencies of muscle biopsy (MB) findings, shown per cluster.

Multimodal integration of clinical and histological data was performed using the nMDS approach [16,17], with k-means clustering applied to data from 64 index cases harboring a variant in *RYR1*. The nMDS method (R^2^ = 0.997) was also used to perform the unsupervised analysis (see Figure S2) and, by means of the elbow method and k-means, we defined a limited set of clusters (k = 4) (see Figure S3). Table S3 shows the distance between the position of the centroids in the four clusters. Figure 4 and Figure 5 provide details of the distribution of HPO codes and the histological findings in the four clusters.

**Figure 6:**
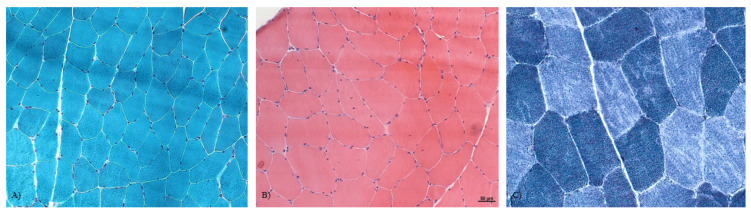
Myopathic changes in patients with *RYR1* alteration. (**A**) Gomori trichrome staining showing some mild variation in fiber size and an increased number of fibers with internal nuclei in Pt 14. (**B**) Hematoxylin and eosin staining demonstrating the variation of fiber sizes, and multiple internal nuclei in some fibers in Pt 12. (**C**) NADH stain showing multiple minicores in some fibers. 40× magnification.

No clear-cut correlation was found between the severity of variants (as measured using CADD scores) and the clusters defined. Cluster 1 was represented by a group of 18 asymptomatic or pauci-symptomatic patients. Two of them presented rhabdomyolysis. Almost all the patients falling in this cluster had undefined myopathic changes, with increased fiber size variation in 11 of them, while 3 cases had internal nuclei, and 2 showed type 1 fiber predominance (Figure 6A, B).

Cluster 2 was formed by a group of 16 patients with clinical findings resembling those of cluster 1 but with more specific and distinctive histological features, including an increase in the number of fibers with internal nuclei in three cases and multiple minicores in one patient (see Figure 6C).

Cluster 3 corresponded to a group of 16 index cases showing proximal muscle weakness of the lower and upper limbs in almost half of the patients and only pelvic muscle weakness in three patients. A distinctive feature of this cluster was the presence of central core myopathy in 37.5% of the patients; moreover, muscle biopsy revealed multiple minicores in 43.7% and increased fiber size variation in 62.5%.

Finally, cluster 4 embraced a group of 14 index cases found to be pauci-symptomatic patients with various combinations of mild weakness and undefined myopathic changes; increased fiber size variation was found in 57.1%. This group of patients also had central cores (n = 8) and multiple minicores (n = 2). Upon WB analyses, significative differences in protein levels were found in cluster 1, and more severely in cluster 4. Nevertheless, variability in the RYR1 protein level among patients that belong to the same cluster was noticed.

Cluster analysis was also applied to sort family groups, and in 14/17 kindred with informative clinical and morphological data, we observed that 10 families (71.4%) belonged to clusters 1 or 2. Asymptomatic or oligosymptomatic recurrent hyperCKemia was found in further members of these 10 families, three of them also showing specific alterations on muscle biopsy (i.e., multiple minicores). A single individual (proband no. 64) reported rhabdomyolysis. Three cluster-3 families (probands no. 54, no. 63, and no. 67, see Table S2) showed mild muscle weakness of the upper and lower limbs; muscle biopsy showed multiple minicores or cores in two of these families. Finally, no families fell into cluster 4.

## 4. Discussion

The molecular diagnosis of inherited skeletal muscle diseases is challenging because of their high clinical and genetic heterogeneity [1,21,22,23]. Even when strict bioinformatic criteria or family segregations are adopted in the diagnostic workup, the level of uncertainty remains high; in particular, uncertainty over the functional consequences of variants (especially missense ones) makes it difficult to predict their pathogenic role. This problem has already been observed with other large muscle proteins, such as titin and nebulin, encoded by the *TTN* and *NEB* genes, respectively, and it seems to be peculiar to “giant” proteins that contribute to the structure of the skeletal muscle membrane. *RYR1* mutation is a common etiology of muscular disorders. In a Spanish study, the molecular etiology was defined in 102 out of 207 patients (49.3%), and the most common causative gene was *RYR1* (followed by *TTN*), accounting for 15% of the cases [1]. A previous investigation in a larger group of 504 subjects found *RYR1* to be the most frequently altered gene (24.3%) among those associated with congenital myopathies [23]. Taken together, these studies (as well as others with similar results) indicate that *RYR1* is among the most common skeletal muscle disease-causing genes [1,23,24,25,26,27].

There has been a recent explosion in the identification of new cases of congenital myopathies due to *RYR1* mutations. Several studies have been published reporting case series carrying various *RYR1* mutations. Some of these have recently identified homogenous groups of patients in a bid to better define specific phenotypes [28,29,30]. In the present study, using extensive clinical, morphological, genetic, and biochemical information from a large cohort of index cases harboring variants in *RYR1*, we attempted an innovative clustering of data to establish more precise correlations and similarities between patients. This was done without choosing any specific clinical or morphological selection criteria a priori. We found that just four clusters were able to group 64 of the patients. Using this approach, we were able to derive the following new information. First, the approach overcame the limits of the “single-dimension” paradigm used for describing “phenotype-gene-protein” relationships. Clustering allowed us to benefit from the innovative, unsupervised strategies more commonly used in the context of complex diseases [31] and, thus, to read multiple data through new scripts integrating clinical features and variants listed as pathogenic, as well as uncover novel information on muscle protein function. Second, this cluster analysis illustrated the limitations of current approaches to clinical phenotyping. Clusters 1 and 2 were highly similar in terms of clinical presentations, even when the symptoms and signs were translated into HPO-based digital codes. Only by combining CADD values with muscle pathology did it prove possible to differentiate between the patients. Third, cluster-based stratification offers new opportunities for better patient care and management. Proximal muscle weakness, occasional exercise intolerance, and myalgia, associated with either malignant hyperthermia and rhabdomyolysis or axial myopathy, were reported across the *RYR1* phenotypes previously described [26]. In particular, myalgia has been reported as almost invariably present in *RYR1*-RM, and often it was the sole presenting feature [27]. The fact that clusters 1 and 2 (which together account for many of our patients) comprised predominantly mild clinical features and subtle histopathological features should be taken as an indication that pauci-symptomatic muscular manifestations should not be dismissed; on the contrary, they require appropriate testing and follow up. Fourth, isolated hyperCKemia is common in *RYR1*-RM patients. About 60% of our index cases presented CK elevation; moreover, high serum CK was detected in more than 50% of the patients in every cluster, even though it was more common in cluster 2. In our clusters, we did not observe many cases with malignant hyperthermia, perhaps because such patients likely escape referral to third-level NMD centers. Malignant hyperthermia should be borne in mind as a possible unfortunate complication of relatively minor muscular manifestations of *RYR1* variants, and these cases, therefore, warrant careful surveillance. Fifth, clustering calls for the adoption of higher-level diagnostic approaches. Clusters 3 and 4 were less represented in the overall sample despite being the ones with the most clearly defined characteristics. Multiple minicores occurred in about 50% of cluster 3 cases and central cores in almost 60% of cluster 4 ones. This indicates that muscle biopsy should always be proposed to individuals harboring mutations in *RYR1* so as to better define their disease manifestations and anticipate clinical trajectories. In detail, from a future perspective, it could be interesting to correlate fiber type composition and levels of RYR1 expression [32]. Indeed, in our cohort, most of the patients with reduced expression of RYR1 had an increased fiber size variation.

That said, we also attempted to apply the same cluster analysis also to *RYR1*-related cohorts with cores described in the recent literature [28,29,30]. Other patients [28,29,30] (Figure S4) with more specific features of core/multicore myopathy were found to fall only within our clusters 3 and, in part, 4. This is not surprising given the more stringent and homogeneous criteria adopted in previous studies. Although our study aimed to offer proof-of-principle evidence, our data need to be interpreted with caution as our multimodal data integration strategies left out about 13% of the whole cohort of *RYR1*-mutated patients. Nonetheless, using a novel mode of data integration, we were able to show that our cohort was comprised mainly of pauci-symptomatic patients with limited muscle weakness and moderately high CK levels against a background of non-specific but at the same time distinctive features of *RYR1*-RM (hyperCKemia, myalgia, cramps with or without mild proximal weakness). Although the presence of such features can be difficult to ascertain in neurological practice, in specialized NMD clinics their detection is crucial both for genetic counseling and for increasing patients’ “safety” during anesthesia. Furthermore, these as well as other clinical aspects can be considered to enrich the power of the clusters and the distance between them. Along the same lines, it might also be useful to consider the full set of genetic data emerging from NGS studies, transcriptomic data deducible from muscle tissue analyses, and the results of calcium imaging to address the issue of protein function in specific variants.

We acknowledge several limitations in our study. First, clinical data and biological samples were not available in some cases because of the retrospective nature of this study. Moreover, WB analyses of muscle biopsy samples were performed only when the amount of muscle was sufficient for the analysis limiting the array of patients where the RYR1 protein was tested. Second, we considered variants with scores > 20 to be pathogenic. This criterion, less stringent than that normally used in similar studies, allowed us to include patients in whom *RYR1* variants may be only part of the genetic background. Indeed, we cannot exclude the possibility that other potential etiologies could have contributed to the muscle features seen in our patients. Third, our model, which sought to find a structure in a collection of interactive data, can be seen as a true data mining challenge. We did not test the robustness of the method with biallelic mutations, and we did not exploit the system with even larger sets of data. Yet, combinations of clinical, morphological, molecular, biochemical, and MRI data are commonly generated in clinical practice and could be adapted to replicate the present methodology with other genes (perhaps *TTN* or *NEB*).

In conclusion, *RYR1* genotype–phenotype correlation is still laborious and often produces poorly defined patterns. Modern assessments might benefit from a higher level of data integration to overcome these limitations. In the era of loose genotype–phenotype correlations, we feel that our approach could be both timely and opportune. Hierarchical and structured clusters of clinical data combined with genetic and morphological variants derived from routine clinical practice could be used to evaluate complex genetic interactions emerging from high-throughput analyses. Were the system to be implemented with additional parameters obtained, for example, through multi-omic analyses in NMDs, it might be possible to fine-tune the difficult process of defining where a single case lies on the spectrum of phenotypes. Translation of this precise patient allocation method to other common NMD-related genes could pave the way not only for more personalized treatment and management approaches but also for the “superclustering” that will be needed to define possible future cocktail therapies. Similar approaches are already paying off in oncogenomic and multifactorial genetic conditions.

## Supplementary Materials

The following supporting information can be downloaded at: https://www.mdpi.com/article/10.3390/genes14020298/s1, Figure S1: Breakdown of muscle strength (measured using MRC scores) in various affected patients at the first and at the last available visit; Figure S2: Stress plot of the nMDS model applied to clinical characteristics and biopsy findings of proband patients; Figure S3: Elbow method: the 4-cluster solution suggested. We used the k-means clustering algorithm and determined the optimal number of clusters by plotting the total within-cluster sum of squares for different numbers of clusters; Figure S4: The cluster analysis applied to *RYR1*-related cohorts with cores described in the recent literature [28,29,30]; Table S1: Clinical, histopathological, and genetic features of the sample. NA= not available, LGMW= limb girdle muscle weakness; Table S2: Clinical, histopathological, and genetic features of the familial cases; Table S3: This table shows the distance of the clusters (in the nMDS model of the patients) from the clusters’ centroids.
