# Supplementary material for: Using Cluster Analysis to Overcome the Limits of Traditional Phenotype–Genotype Correlations: The Example of RYR1-Related Myopathies
Source: Genes (Basel). 2023 Jan 23;14(2):298. doi: 10.3390/genes14020298 (PMC9956305; doi:10.3390/genes14020298)
Supplement: Supplementary file 1 [file genes-14-00298-s001.zip › Legend to Supplementary material.pdf]

Legend to Supplemental material

Figure S1: Breakdown of muscle strength (measured using MRC scores) in various affected patients at the first and at the last available visit.

Figure S2: Stress plot of the nMDS model applied to clinical characteristics and biopsy findings of proband patients. The stress plot is a Shepard plot where ordination distances are plotted against the original sample's dissimilarities and the fit is shown as a monotone step line. The figure also shows two correlations as statistics of goodness of fit. The correlation based on stress (non-metric fit) is $R^2 = 1 - S^2$, where S is the final stress value of the model. The fit-based R^2 is the correlation between the fitted values and ordination distances (linear fit).

Figure S3: Elbow method: the 4-cluster solution suggested. We used the k-means clustering algorithm and determined the optimal number of clusters by plotting the total within-cluster sum of squares for different numbers of clusters. The location of a bend (knee) in the plot is generally considered an indicator of the appropriate number of clusters.

Figure S4: The cluster analysis applied to RYR1-related cohorts with cores described in the recent literature [28−30].

Table S1: Clinical, histopathological, and genetic features of the sample. NA= not available, LGMW= limb girdle muscle weakness

Table S2: Clinical, histopathological, and genetic features of the familial cases. f=father; m=mother; s=son; si=sister, c=cousin, NA=not available, AD=autosomal dominant, n.k.=family relationship not known

Table S3: This table shows the distance of the clusters (in the nMDS model of the patients) from the clusters' centroids.
