# Supplementary figures and images for: Using Cluster Analysis to Overcome the Limits of Traditional Phenotype–Genotype Correlations: The Example of RYR1-Related Myopathies
Source: Genes (Basel). 2023 Jan 23;14(2):298. doi: 10.3390/genes14020298 (PMC9956305; doi:10.3390/genes14020298)

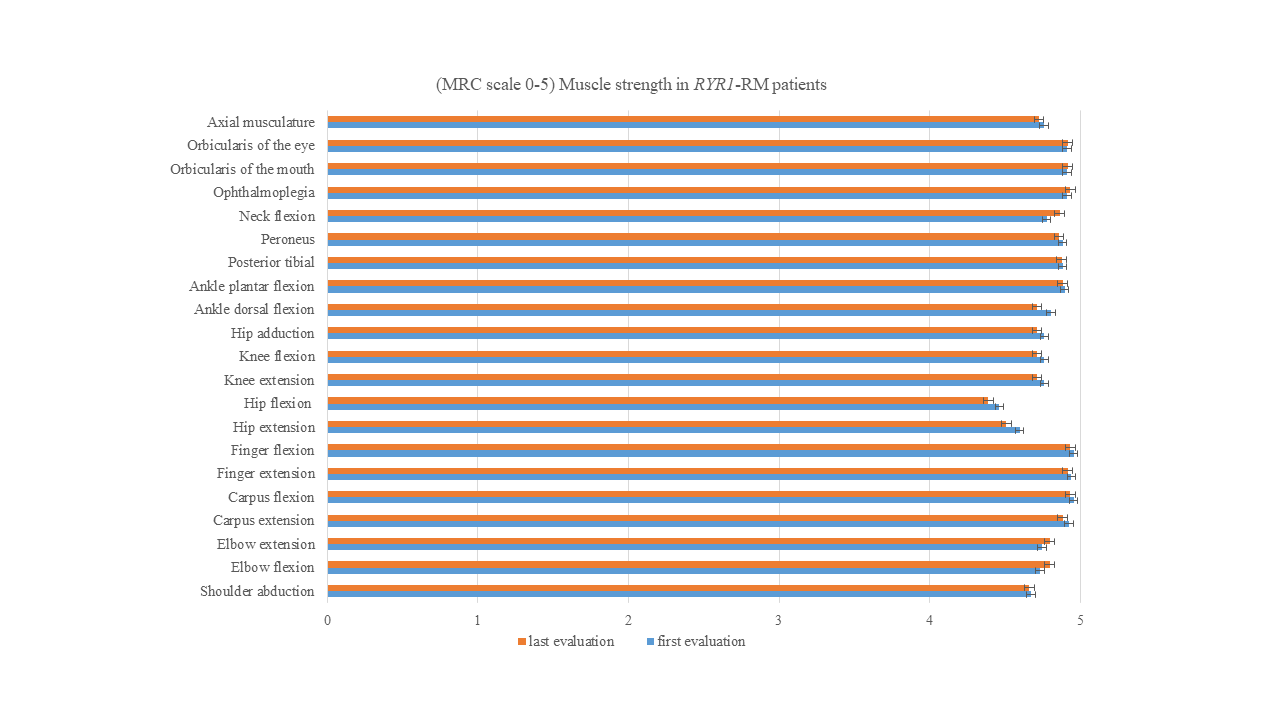

Supplement: Supplementary file 1 [file genes-14-00298-s001.zip › Figure S1 new.tif]

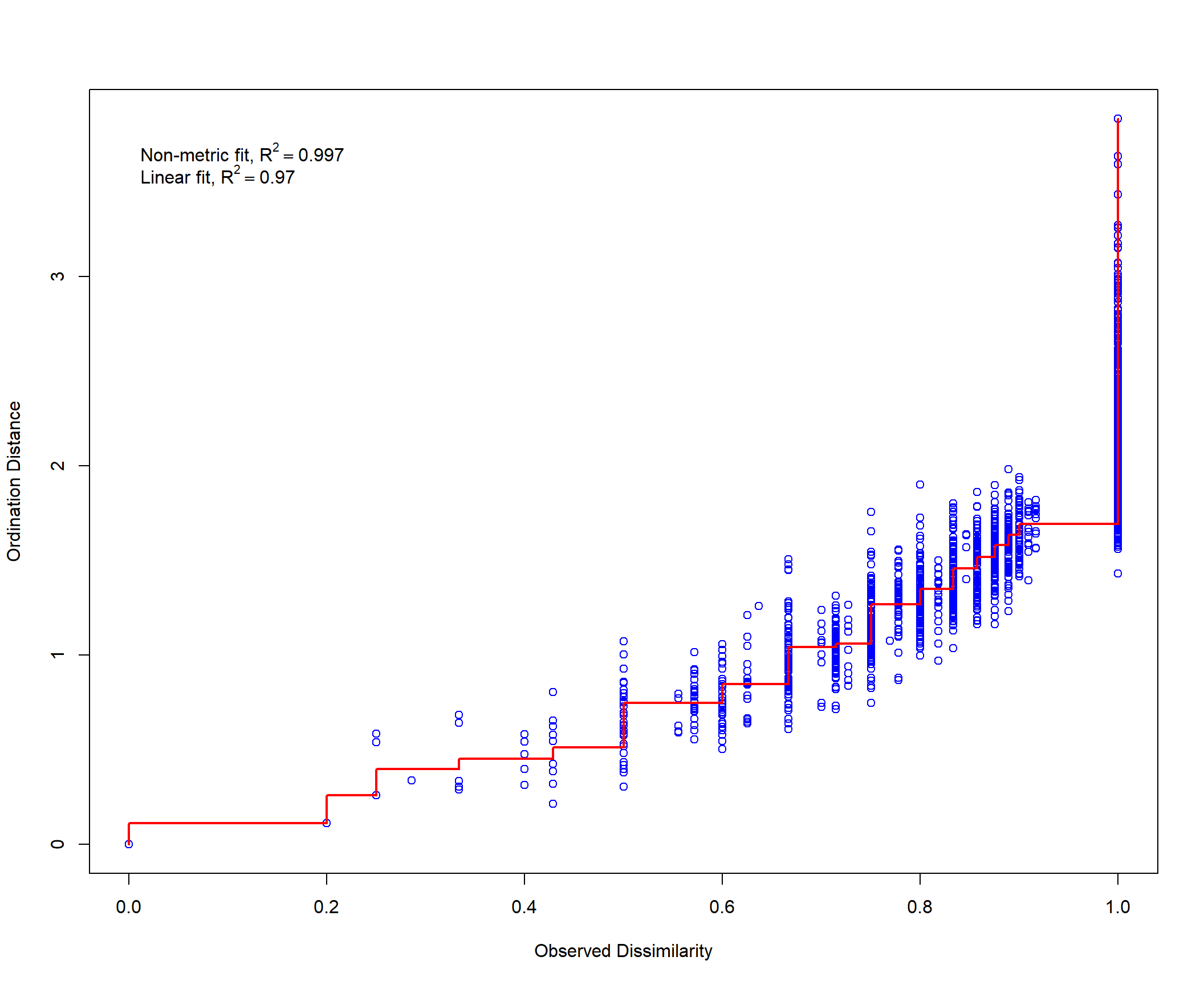

Supplement: Supplementary file 1 [file genes-14-00298-s001.zip › Figure S2.png]

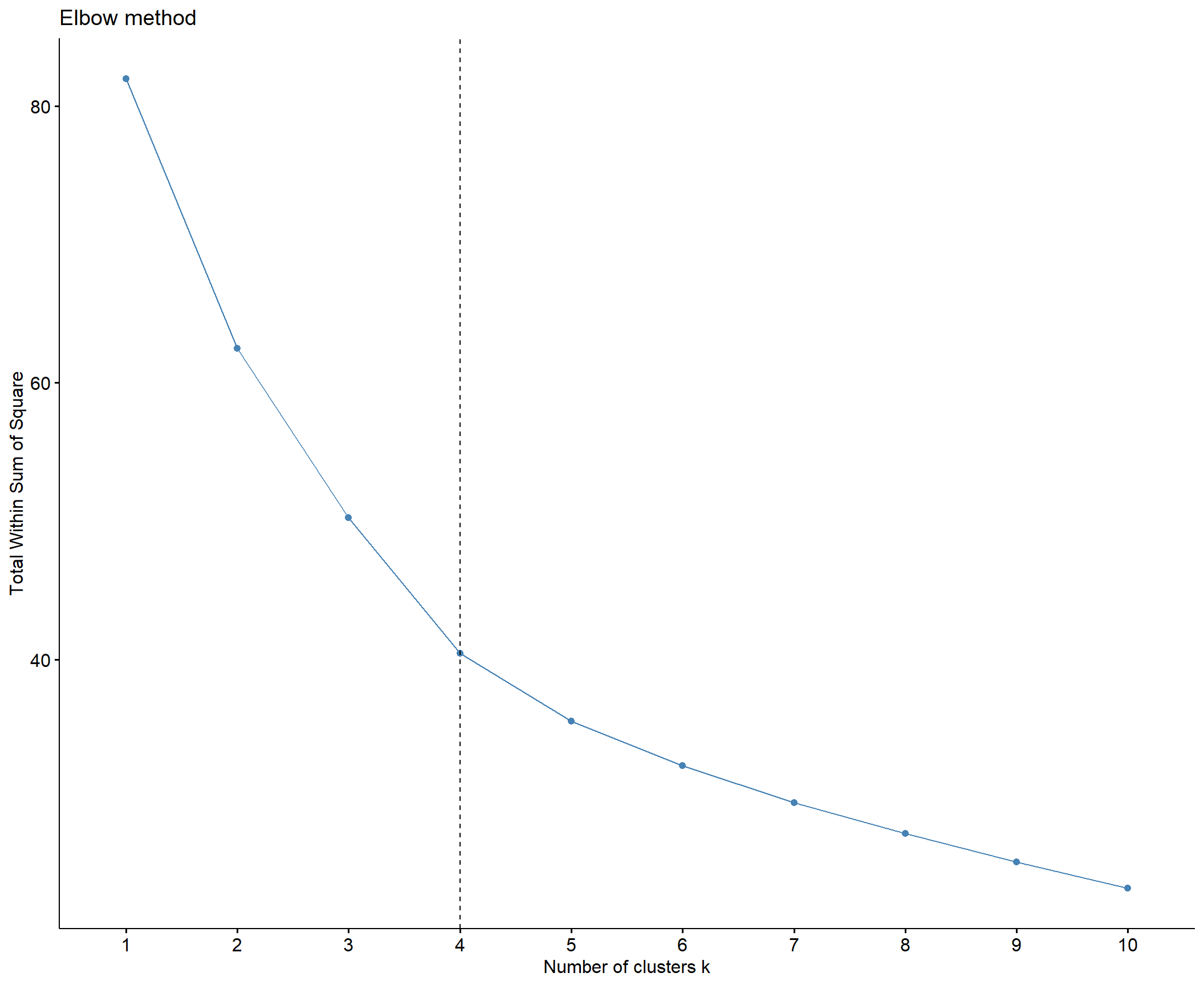

Supplement: Supplementary file 1 [file genes-14-00298-s001.zip › Figure S3.png]

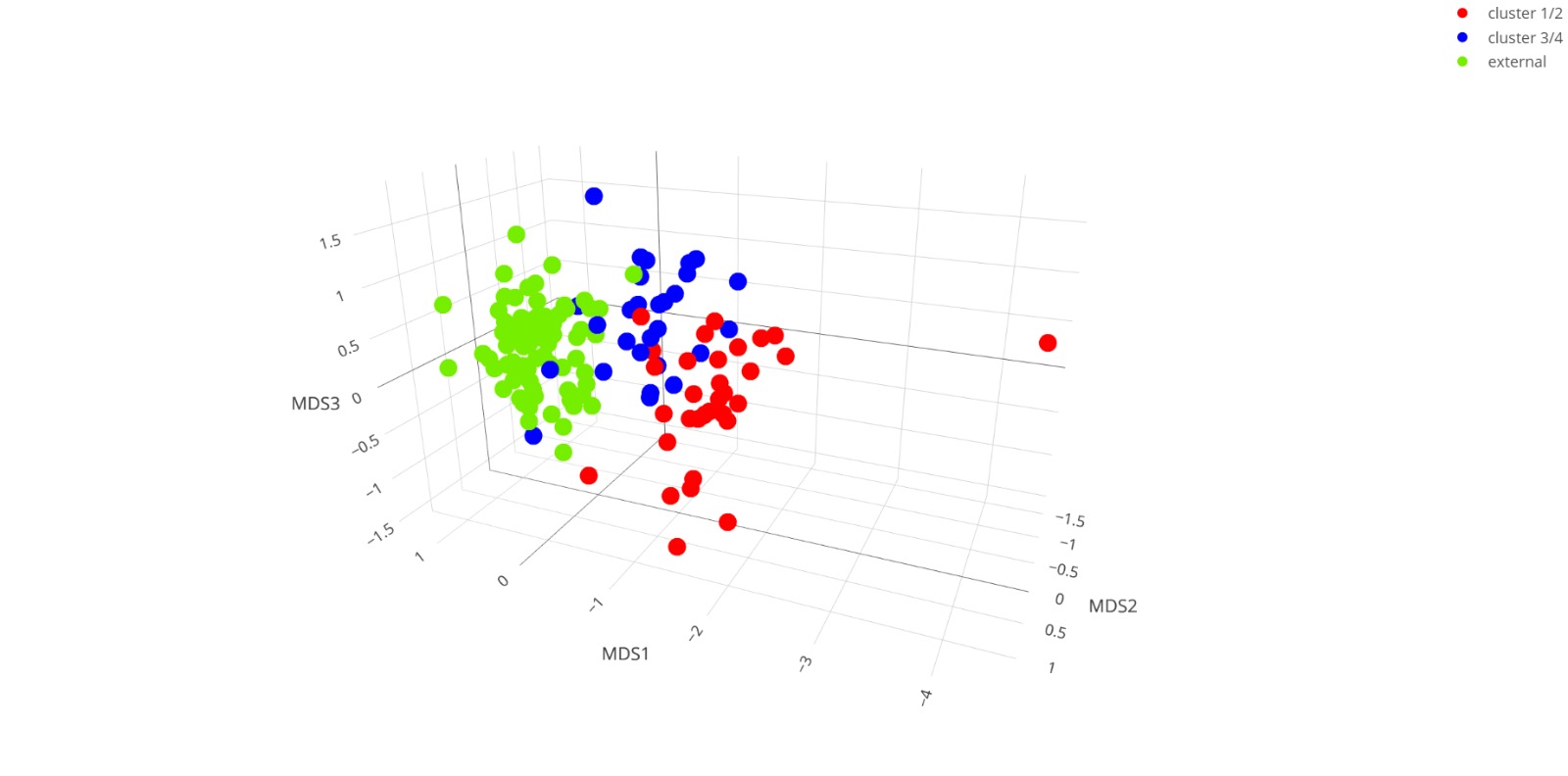

Supplement: Supplementary file 1 [file genes-14-00298-s001.zip › Figure S4.jpeg]

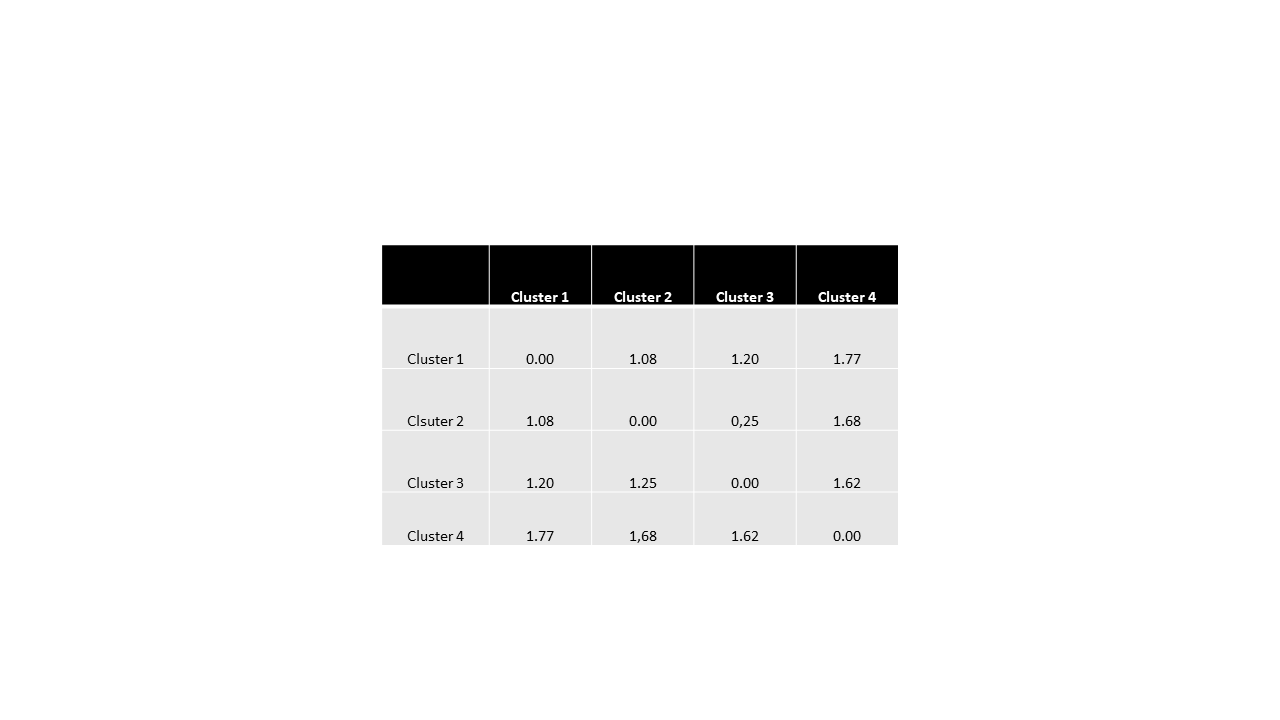

Supplement: Supplementary file 1 [file genes-14-00298-s001.zip › Table S3.tif]
